# Deep learning-based detection and quantification of brain metastases on black-blood imaging can provide treatment suggestions: a clinical cohort study

**DOI:** 10.1007/s00330-023-10120-5

**Published:** 2023-09-02

**Authors:** Hana Jeong, Ji Eun Park, NakYoung Kim, Shin-Kyo Yoon, Ho Sung Kim

**Affiliations:** 1 Department of Radiology and Research Institute of Radiology, University of Ulsan College of Medicine, Asan Medical Center, 43 Olympic-ro 88, Songpa-Gu, 05505 Seoul, Korea; 2 Dynapex LLC, Seoul, South Korea; 3 Department of Oncology, Asan Medical Center, Seoul, South Korea

**Keywords:** Deep learning, Brain metastases, Treatment, Suggestion

## Abstract

**Objectives:**

We aimed to evaluate whether deep learning–based detection and quantification of brain metastasis (BM) may suggest treatment options for patients with BMs.

**Methods:**

The deep learning system (DLS) for detection and quantification of BM was developed in 193 patients and applied to 112 patients that were newly detected on black-blood contrast-enhanced T1-weighted imaging. Patients were assigned to one of 3 treatment suggestion groups according to the European Association of Neuro-Oncology (EANO)-European Society for Medical Oncology (ESMO) recommendations using number and volume of the BMs detected by the DLS: short-term imaging follow-up without treatment (group A), surgery or stereotactic radiosurgery (limited BM, group B), or whole-brain radiotherapy or systemic chemotherapy (extensive BM, group C). The concordance between the DLS-based groups and clinical decisions was analyzed with or without consideration of targeted agents. The performance of distinguishing high-risk (B + C) was calculated.

**Results:**

Among 112 patients (mean age 64.3 years, 63 men), group C had the largest number and volume of BM, followed by group B (4.4 and 851.6 mm^3^) and A (1.5 and 15.5 mm^3^). The DLS-based groups were concordant with the actual clinical decisions, with an accuracy of 76.8% (86 of 112). Modified accuracy considering targeted agents was 81.3% (91 of 112). The DLS showed 95% (82/86) sensitivity and 81% (21/26) specificity for distinguishing the high risk.

**Conclusion:**

DLS-based detection and quantification of BM have the potential to be helpful in the determination of treatment options for both low- and high-risk groups of limited and extensive BMs.

**Clinical relevance statement:**

For patients with newly diagnosed brain metastasis, deep learning–based detection and quantification may be used in clinical settings where prompt and accurate treatment decisions are required, which can lead to better patient outcomes.

**Key Points:**

• *Deep learning–based brain metastasis detection and quantification showed excellent agreement with ground-truth classifications.*

• *By setting an algorithm to suggest treatment based on the number and volume of brain metastases detected by the deep learning system, the concordance was 81.3%.*

• *When dividing patients into low- and high-risk groups, the sensitivity for detecting the latter was 95%.*

**Supplementary Information:**

The online version contains supplementary material available at 10.1007/s00330-023-10120-5.

## Introduction

With advances in cancer treatment, the survival of cancer patients is improving, but this improved survival leads to an increasing proportion being diagnosed with brain metastasis (BM) [1]. Accurate assessment of BM is necessary for disease monitoring and response assessment in clinical practice. Nonetheless, the detection of BMs can be rather laborious for radiologists, and is often limited in that segmentation and quantification of BMs are frequently not provided when MRI is read [2].

The feasibility of deep learning systems (DLSs) for the automatic detection of BM has been well demonstrated [1, 3–8]. Most DLSs have used object detection techniques and marked the presence of BMs using a bounding box [1, 8], rather than performing segmentation and quantification of BMs. If voxel-wise segmentation is used for DLS-based BM detection, accurate volumetry should be possible, and the clinical utility of the system should be higher. Black-blood (3D turbo-spin echo) contrast-enhanced T1-weighted imaging (CE-T1WI) was recently proposed as a recommended protocol in clinical trials [9], which reduces enhancing vessels mimicking brain metastasis [10–12]. Compared with DLS using only gradient-echo CE-T1WI, that using a combination of gradient-echo and black-blood CE-T1WI showed high sensitivity of 82.4% for detecting BMs smaller than 3 mm, with a substantially lower false-positive rate of 0.59 per patient [13]. However, studies on the clinical utility of DLS incorporating black-blood CE-T1WI for the detection and quantification of BMs are lacking.

According to the American Society of Clinical Oncology (ASCO), the Society for Neuro-Oncology (SNO), the American Society for Radiation Oncology (ASTRO) practice guidelines [14], and the European Association of Neuro-Oncology (EANO)-European Society for Medical Oncology (ESMO) recommendations for BM from solid tumors [15], the volume and number of BMs and the patient’s condition should be taken into account when deciding on treatment. The treatment suggestions include stereotactic radiosurgery (SRS), surgery, whole-brain radiotherapy (WBRT), and systemic chemotherapy [15]. In this regard, the role of SRS for BM has evolved [16], and patients with up to ten BMs can be indicated for SRS. Because numeric and volume-based evaluations are important in treatment planning, we hypothesized that deep learning-based detection and quantification could assist in providing treatment suggestions for BM. Therefore, the purpose of this study was to evaluate whether deep learning-based detection and quantification of BM may suggest treatment options for patients with BMs.

## Materials and methods

### Eligibility criteria

**Fig. 1:**
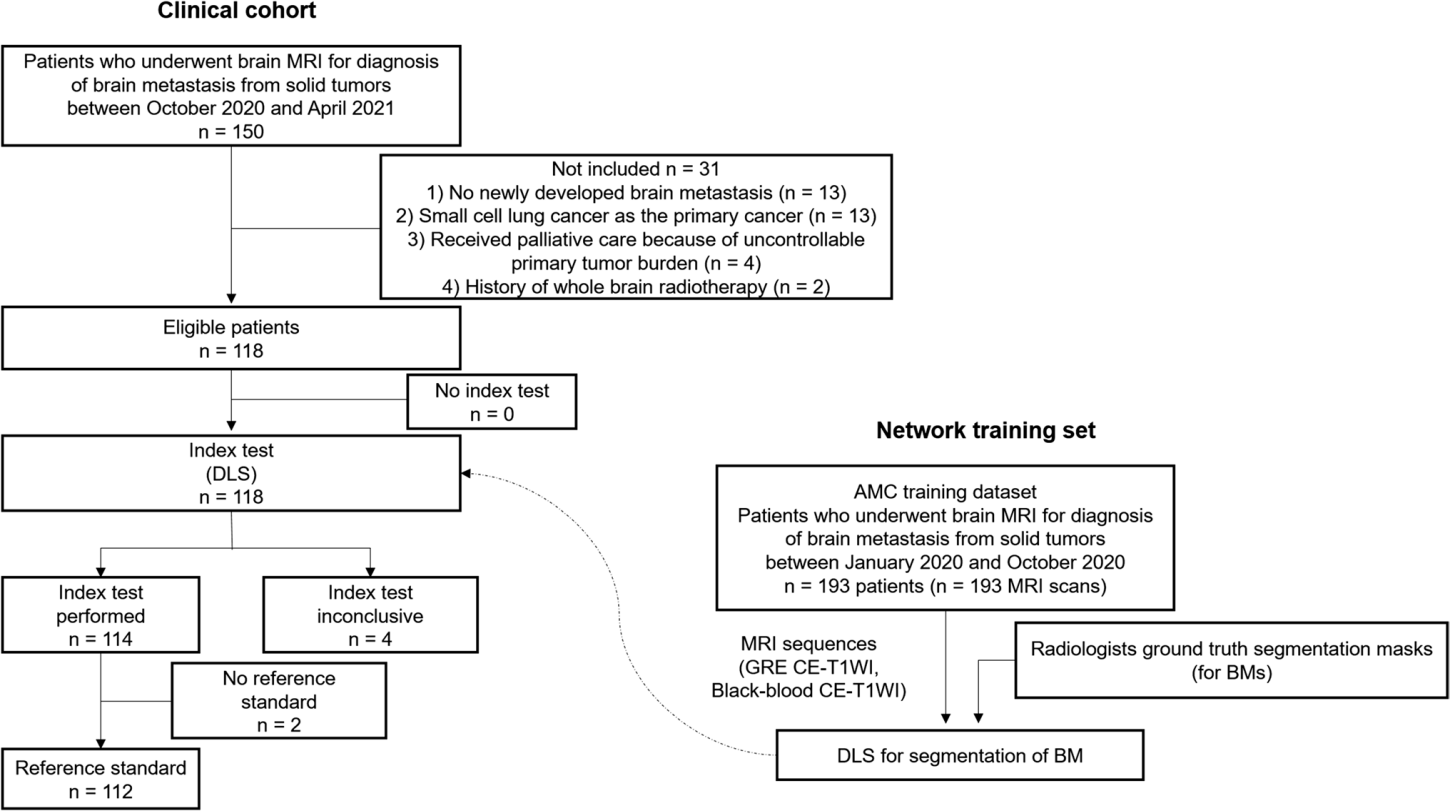
Patient inclusion process for the clinical cohort and network training set. DLS = deep learning system, BM = brain metastasis, GRE = gradient echo, CE-T1WI = contrast-enhanced T1-weighted imaging

This study was conducted in accordance with the Declaration of Helsinki, and the study protocol was approved by the institutional review board of Asan Medical Center (IRB number: 2021–0963). The requirement for informed consent was waived because of the retrospective nature of the study. The patient inclusion process is shown in Fig. 1. A clinical cohort of 150 consecutive patients who underwent brain MRI for diagnosis of BM from solid tumors between October 2020 and April 2021 were screened. Patients were excluded if (1) they did not have newly developed BM (*n* = 13), (2) their primary tumor was a small cell lung cancer (*n* = 13; because the decision to perform SRS or WBRT and the timing of such interventions differ from those of solid tumors) [17], (3) they underwent palliative therapy because of an uncontrollable primary tumor burden (*n* = 4), or (4) they had history of previous whole-brain radiotherapy (*n* = 2). Six additional patients were excluded, either because of an inconclusive index test with the presence of enhancing brain lesion other than BM (*n* = 4) or because the reference standard follow-up was missing (*n* = 2). Finally, 112 patients were included in the analysis (mean [standard deviation (SD)] age, 64.3 [9.8] years; 63 men).

The deep learning network for detection and quantification of BM was developed on a separate training dataset of 193 patients, of whom 93 had BM (866 BMs) and 100 were negative for BM. These 193 patients underwent brain MRI between January 2020 and October 2020. The distribution of BMs, distribution of primary tumors, and performance of the DLS in the training set are summarized in Supplementary Table 1 and 2.

### MRI acquisition protocols

The BM imaging protocol was acquired using a 3-T scanner with either a 32-channel or 64-channel head coil (Ingenia 3.0 CX, Philips Healthcare; Architect, GE Healthcare; Vida, Siemens Healthineers). A detailed image protocol is in the supplementary material.

### DLS for detection and quantification of BM

In the training of our DLS model, a GRE CE-T1WI and black-blood CE-T1WI image pair was fed into the model as input. Details of the DLS are summarized in the supplementary material. The network design is shown in Supplementary Fig. 1.

### Reference tumor volume for DLS output

The ground-truth volumes were separately prepared by two researchers (M.S.K. and H.J.K., with 7 and 2 years of experience in radiology, respectively) using co-registered GRE CE-T1WI and black-blood CE-T1WI. The readers simultaneously reviewed both images and drew ground-truth volume boundaries on black-blood CE-T1WI. The BMs detected by the DLS were considered true-positive findings if they had at least one voxel overlapping with a ground-truth volume and false-positive findings if they had no voxels overlapping with any of the ground-truth volumes.

### Suggestion of treatment according to the DLS output

**Fig. 2:**
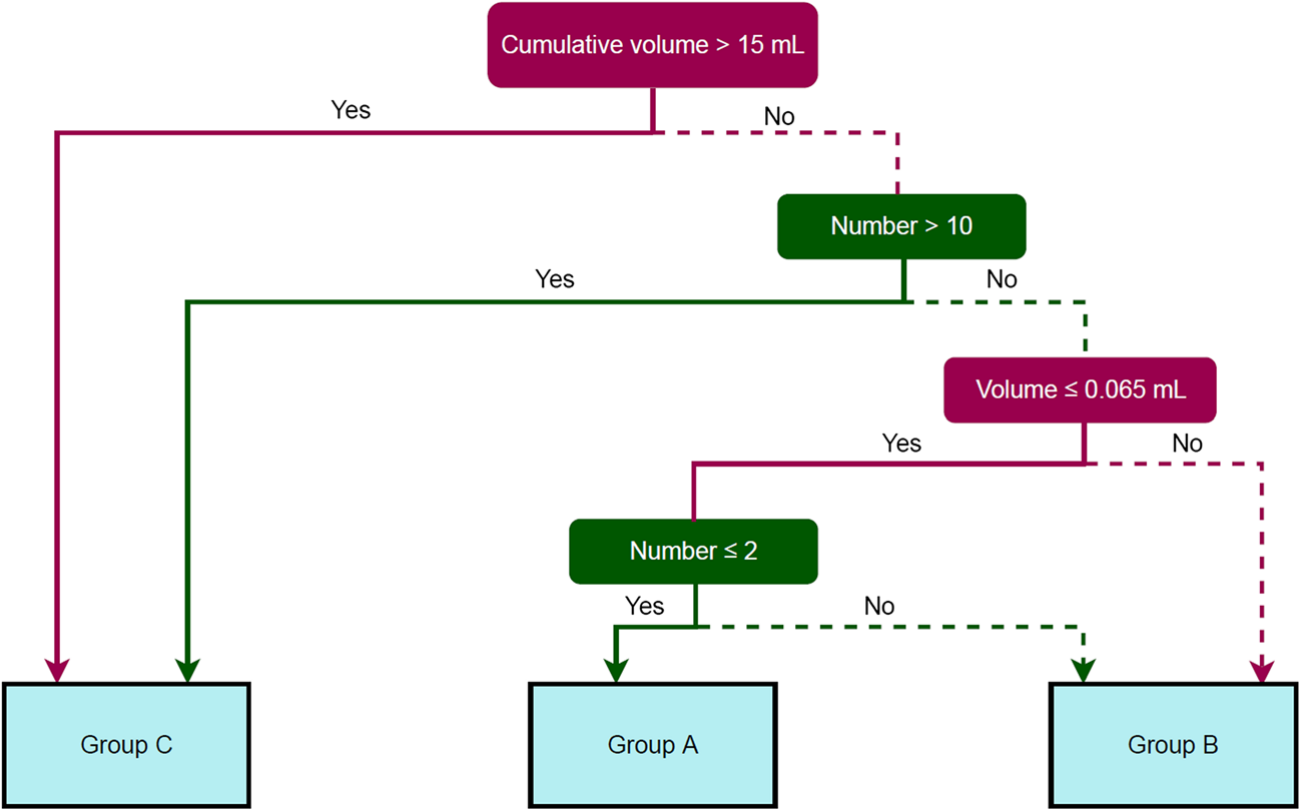
The logical algorithm for assignment of treatment options for newly developed brain metastasis (BM) according to deep-learning system output

To suggest treatment according to the number and volume of BMs detected by the DLS, we set a logical algorithm for assignment of treatment suggestions on the basis of the combination of different treatment approaches proposed by the ASCO-SNO-ASTRO [14] and EANO-ESMO practice guidelines [15]. A diagram summarizing the practice guidelines is presented in Fig. 2. We defined three treatment groups according to the number and volume of the BMs in the DLS output.

- Group A (indicated for follow-up): patients with new brain lesions smaller than 5 mm are considered “not measurable” on MRI with 1.5-mm slice thickness or less according to the response assessment in neuro-oncology BM (RANO-BM) [17]. A follow-up MRI scan is needed to identify whether such small lesions are true metastasis [18]. The patients in group A had ≤ 2 BMs with a diameter ≤ 5 mm and calculated volume ≤ 65 mm^3^ on DLS output, and were recommended for short-term imaging follow-up without treatment. The rationale for a calculated volume of 65 mm^3^ was based on the formula for the volume of a sphere, ${\text{V}} \, \text{=}\frac{4}{{3}}\pi {\text{r}}^{3}$ (*V*: volume, *r*: radius), with 5 mm as the largest diameter of a given lesion.
- Group B (limited BMs indicated for surgery or SRS): surgery or SRS is recommended for patients with a single brain lesion or several lesions (equal to or less than ten with a cumulative volume less than 15 mL [17]). Group B was defined as those patients recommended for surgery or stereotactic radiosurgery with a total tumor volume > 65 mm^3^ or lesion count ≥ 3 but with lesion count and volume not more than 10 and 15 mL (15,000 ${\text{mm}}^{3}$), respectively.
- Group C (extensive BMs with chemotherapy or WBRT recommended): patients with more than ten BMs and uncontrolled extra-CNS disease should be considered for WBRT or systemic chemotherapy [15]. Extensive BM was defined as several lesions (≤ 10) with a volume > 15 mL (15,000 ${\text{mm}}^{3}$) or more than ten lesions regardless of total volume.

### Updated treatment suggestion considering targeted agents

Recently, with the development of various targeted therapies in molecular mutation, there have been many changes in the treatment of cancer, especially non-small cell lung cancer (NSCLC) [19]. If NSCLC patients with BM have molecular mutations in epidermal growth factor receptor (EGFR) or anaplastic lymphoma kinase (ALK), upfront chemotherapy using targeted agent, osimertinib or alectinib, is recommended as first-line therapy instead of local therapy such as surgery or SRS unless there are symptoms related to BM [20]. Since the DLS-based treatment suggestion did not consider this molecular information, in patients who were suggested as “group B” by DLS but whose actual treatment was upfront chemotherapy, we regarded it correct according to the current guideline and obtained modified treatment suggestion.

### Reference standards for treatment suggestion

Reference standard treatment suggestions were made for each patient on the basis of their medical charts by one of the authors (H.N.J., with 3 years of experience in radiology) who was not involved in the image analysis and consulted with an oncologist (Y.K.S., with 15 years of experience in oncology).

The reference standard was achieved by checking the patient’s clinical records to identify which treatment the patient actually underwent. The patient treatment suggestions for newly developed BM were summarized as follows: (1) patients did not change therapy and underwent follow-up brain MRI within 3 months, (2) patients underwent surgery, (3) patients underwent SRS, (4) patients underwent systemic chemotherapy, and (5) patients underwent WBRT.

### Statistical analysis

#### Detection performance of the DLS

To evaluate the performance of the DLS for BM detection, the results were compared according to the number of BMs. Sensitivity was defined as the ratio between the number of true-positive findings and all metastases in the entire testing set (true-positive findings/[true-positive findings + false-negative findings]). Positive predictive value (PPV) was defined as the ratio between the number of true-positive findings and all detections (true-positive findings/[true-positive findings + false-positive findings]). True-positive findings reflect the number of metastases correctly detected and identified as metastasis. False-negative findings reflect the number of metastases incorrectly not identified as metastasis. False-positive findings reflect the number of non-metastases incorrectly identified as metastasis. A free-response receiver operating characteristic curve was also calculated for all lesions and all patients.

#### Performance of the DLS in BM quantification

Agreement for the numbers and volumes of detected BMs between the DLS and ground-truth data was calculated for the clinical cohort using the intraclass correlation coefficient (ICC), with a two-way mixed model with consistency type.

#### Characteristics of the treatment groups

The Kruskal–Wallis test was used to evaluate the differences in BM numbers and volume between each group, and this analysis was complemented by post hoc analysis using the Bonferroni correction and Mann–Whitney *U* test.

#### Accuracy of treatment suggestions

The DLS-based treatment suggestions were considered correct if they were identical to the reference standard patient treatments, and accuracy was defined as the percentage of treatment suggestions identical to the clinician’s decisions. The accuracy, sensitivity, and specificity for correct treatment suggestions were defined as follows:

$${\text{Accuracy}} \, \text{=} \, \frac{{\text{TP}} \, \text{+} \, {\text{TN}}}{{\text{TP}} \, \text{+} \, {\text{TN}} \, \text{+} \, {\text{FP}} \, \text{+} \, {\text{FN}}}\text{, Sensitivity} \, \text{=} \, \frac{\text{TP}}{{\text{TP}} \, \text{+} \, {\text{FN}}}\text{, Specificity} \, \text{=} \, \frac{\text{TN}}{{\text{TN}} \, \text{+} \, {\text{FP}}}$$

The DLS-based treatment suggestions were tested both with and without consideration of targeted agents. Then, the ground-truth volume-based treatment suggestions were compared with the DLS-based treatment suggestions.

Concordance was also evaluated according to the weighted kappa between the DLS-based treatments and actual clinical decisions. A *p* value < 0.05 was considered to represent a statistically significant difference. SPSS (version 21) was used for all statistical analyses.

## Results

### Characteristics of the patients and BMs in the clinical cohort

One hundred and twelve patients (mean age ± SD, 64.3 ± 9.8 years, 63 men [56%]) were enrolled as the study population, and their clinical characteristics are summarized in Table 1. Lung was the most common primary site of cancer (81.3% [91 of 112]), followed by other types (prostatic cancer, genitourinary cancer) of cancer (8.9% [10 of 112]), breast cancer (3.6% [4 of 112]), and renal cell cancer (3.6% [4 of 112]). Among the lung cancers, adenocarcinoma was the most common type (91.2% [83 of 91]), followed by squamous cell carcinoma (6.6% [6 of 91]).

**Table 1:** Baseline characteristics of the clinical cohort

Characteristics	
Number of patients	112
Age (years)*	64.3 ± 9.8
Gender (male to female)	63:49
Cancer type (%)	
Lung (NSCLC)	91 (81.3)
Breast	4 (3.6)
Colorectal	3 (2.7)
Renal	4 (3.6)
Others	10 (8.9)
Number of BMs	952
Average number of BMs per patient	8.5 ± 12.22
Patients with > 10 BMs	25 (22.3)
Volume (mm^3^) of BM	
Mean ± SD	1814.5 ± 5115.5
Number of BMs < 65 mm^3^	691 (72.6)
Treatment (%) after diagnostic imaging	
Short-term follow-up without treatment	23 (20.5)
SRS	56 (50.0)
Surgery	1 (0.9)
WBRT	11 (9.8)
Systemic chemotherapy	21 (18.8)

*BM* brain metastasis, *WBRT* whole-brain radiotherapy

^*^Continuous variables are expressed as mean ± standard deviation. Numbers in parentheses are percentages

A total of 952 BMs were detected in the 112 patients. The mean number of BMs per patient was 8.5 (SD, 12.22), and the mean volume was 1814.5 mm^3^ (SD, 5115.5 mm^3^). Of the 952 BMs, 691 (72.6%) were smaller than 65 mm^3^. The distributions of patients and BMs are shown in Supplementary Fig. 2.

### Performance of DLS in the detection and quantification of BMs

In the clinical cohort, the detection performance showed a sensitivity of 91.3% (876 of952) and PPV of 84.5% (876 of 1036). The free-response ROC curve is shown in Supplementary Fig. 3. There was excellent agreement in BM numbers between the DLS output and ground-truth data (ICC, 0.986; 95% confidence interval [CI] 0.979–0.990). There were 160 false-positive findings among the 1036 lesion detections, giving a rate of 0.70 per patient. Frequent patterns of false-positive and false-negative findings are summarized in Supplementary Table 3 and Supplementary Fig. 4. The volume measurements also showed excellent agreement between the DLS output and ground-truth data (ICC, 0.893; 95% CI 0.845–0.927).

### Characteristics of the treatment groups

Table 2 shows the differences in the total number and volume of the BMs according to the three DLS-based groups. The DLS most commonly assigned patients to group B (60 patients, 54%; patients with limited BMs recommended for surgery or stereotactic radiosurgery), followed by groups A and C. Group C showed the largest numbers and volume, followed by groups B and A (Kruskal–Wallis test, *p* < 0.001 and *p* = 0.001, respectively).

**Table 2:** Differences between treatment groups A, B, and C allocated by the deep-learning system

	Group A	Group B	Group C	*p* value
Number of patients	26	60	26	
Mean number of lesions	1.46 (1–2)	4.42 (1–10)	27.96 (7–91)	< .001
Mean total volume (mm^3^)	15.54 (3–63)	851.58 (12–11,471)	3805.92 (403–24,602)	< .001

Numbers in parentheses are ranges. Group A: short-term imaging follow-up without treatment; Group B: limited brain metastasis with surgery or stereotactic radiosurgery; Group C: extensive brain metastasis with whole-brain radiotherapy or systemic chemotherapy

### Accuracy of treatment suggestions

**Fig. 3:**
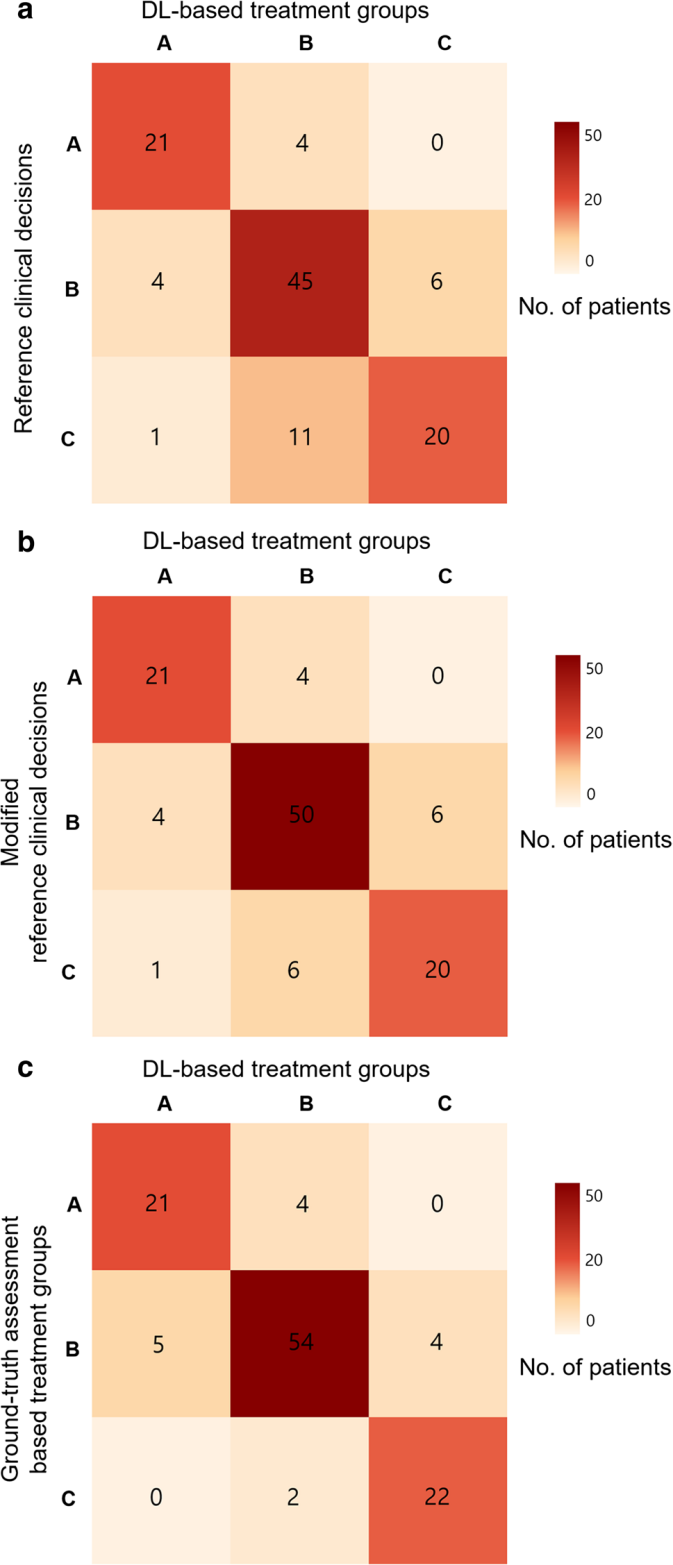
The resulting patient treatment suggestions The confusion matrix displays the number of patients recommended for each treatment option by the deep learning system (DLS) and how they compare to the clinical decisions. The DLS-based treatment suggestions are represented on the *x*-axis, while the *y*-axis denotes the actual clinical decision groups (**a**), modified clinical decision groups (**b**), or ground-truth assessment-based treatment groups (**c**). The numbers of correct decisions are found on the diagonal

Figure 3a presents a confusion matrix of the patient numbers and treatment suggestions by the DLS and the actual clinical decisions. The DLS-based treatment suggestions correctly assigned 76.8% (86 of 112) of patients. Eleven patients (9.8%) were classified into group B by the DLS but to group C according to the actual clinical decision, with this classification disagreement being the most frequent case among the 26 patients showing disagreement.

**Fig. 4:**
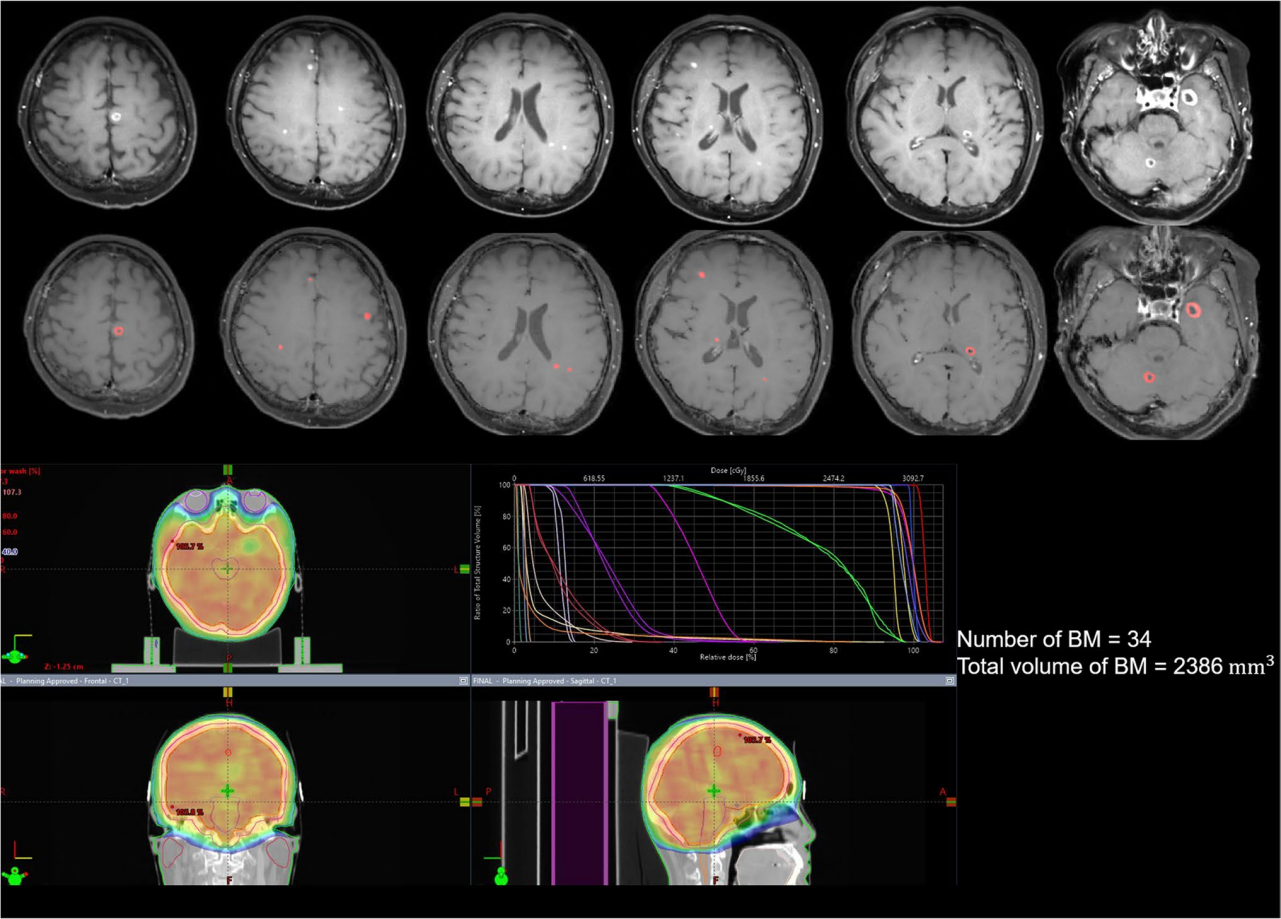
A 50-year-old woman with lung cancer (adenocarcinoma) showed multiple BMs on brain MRI. According to the DLS, there were 34 BMs with a total volume of 2386 mm^3^; thus, she was assigned to the extensive BM group. A few days later, the patient received WBRT

**Fig. 5:**
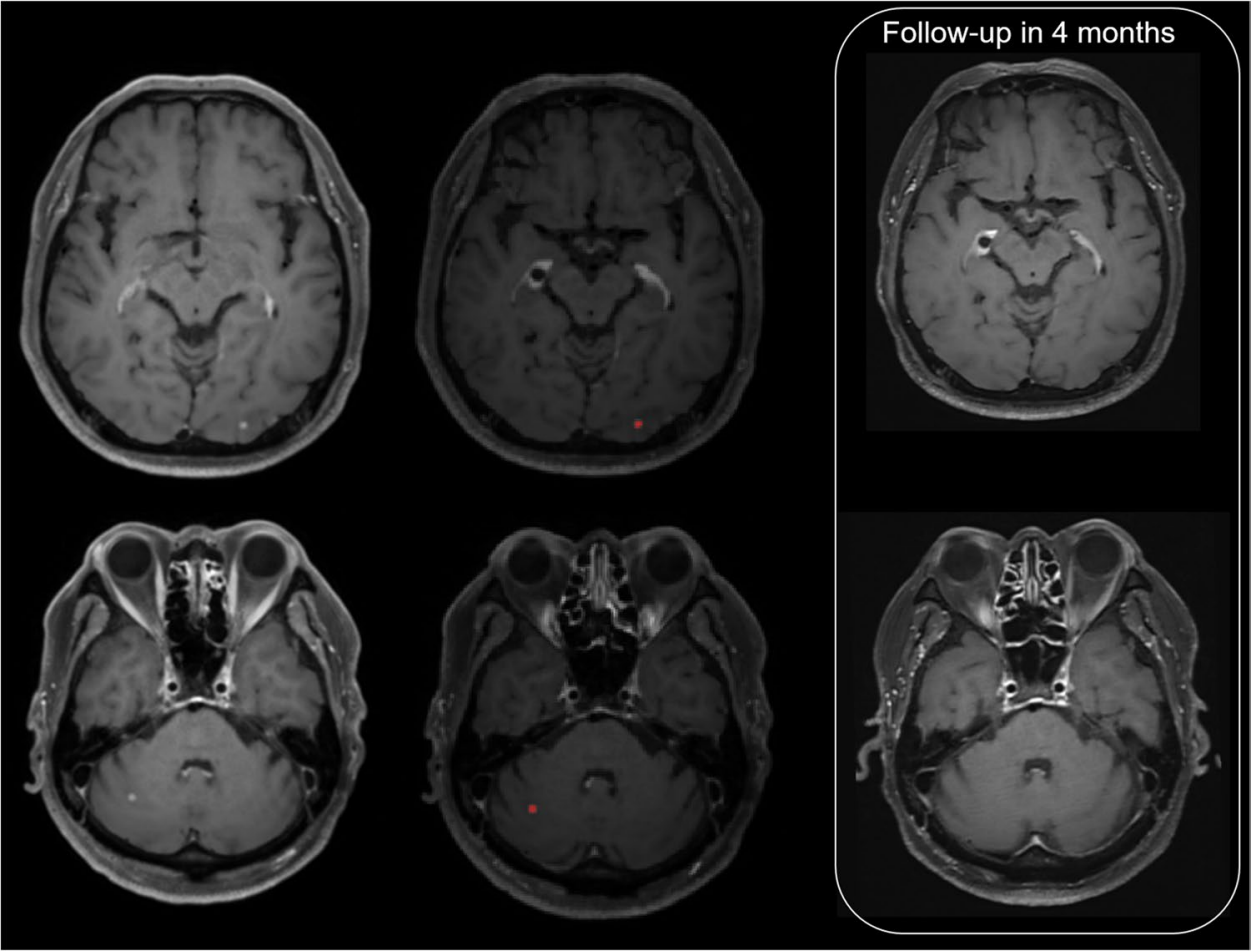
A 65-year-old man with lung cancer (adenocarcinoma) showed a few tiny enhancing lesions on brain MRI. The DLS detected two lesions with a total volume of 22 mm^3^, and the patient was therefore assigned to the follow-up group. On the follow-up MRI conducted 4 months later, there were no definite enhancing lesions in brain parenchyma

The DLS showed diagnostic sensitivity of 84% (21 of 25) for assignment to group A, 81.8% (45 of 55) for assignment to group B, and 62.5% (20 of 32) for assignment to group C. Relevant cases are shown in Figs. 4 and 5.

Although there were several cases in which the treatment suggestions by the DLS differed from actual clinical decisions, most of them were acceptable considering the various clinical situations that occurred. Supplementary Table 4 summarizes the cases with discrepancies, and the discrepant cases are explained in the supplementary material.

### Accuracy of modified treatment suggestions considering targeted agents

Figure 3b presents a confusion matrix of patient numbers and treatment suggestions by the DLS and modified actual clinical decisions. In newly diagnosed NSCLC patients with EGFR or ALK mutation, five patients received upfront chemotherapy instead of local therapy. We obtained the modified accuracy by considering that the five patients were actually the same as those who received local therapy such as surgery or SRS. In this way, the DLS-based treatment suggestion showed an accuracy of 81.3% (91 of 112).

### Comparison between DLS-based treatment suggestions and ground-truth volume-based treatment suggestions

Figure 3c presents a confusion matrix of patient numbers and treatment suggestions according to the DLS output and ground-truth data using the same algorithm. The concordance rate for the treatment suggestions obtained using the two data sets reached 86.6% (97 of 112), demonstrating objectively high agreement.

### Sensitivity and specificity for distinguishing high-risk patients using accuracy of modified treatment suggestions

Since group A was a follow-up group without any treatment, we refer to it as low risk. By contrast, patients in groups B and C required treatment, and we classified them as high risk. The sensitivity and specificity for DLS detection of high-risk patients were 95% (82 of 86) and 81% (21 of 26), respectively. The sensitivity and specificity for distinguishing high-risk patients were the same, whether or not considering modified treatment suggestions.

## Discussion

In our study, we investigated whether deep learning-based detection and quantification of BM could suggest treatment options for patients with BMs. By setting a logical algorithm to suggest treatment according to the number and volume of BMs detected by the DLS in accord with recent guidelines from the ASCO-SNO-ASTRO [14] and EANO-ESMO [15], treatment suggestions showing 76.8% (86 of 112) concordance with the actual clinical decisions were attained. When considering upfront chemotherapy as separate treatment and calculating modified accuracy, it showed accuracy of 81.3% (91 of 112). In addition, when we divided the treatment suggestions into the two low-risk and high-risk groups according to whether the patients needed follow-up or any treatment, the sensitivity and specificity for detecting the high-risk group were 95% (82 of 86) and 81% (21 of 26), respectively. Thus, our study demonstrated that DLS-based detection and quantification of BMs can provide treatment suggestions showing considerable accuracy in comparison with actual clinical decisions.

The purpose of our study was twofold: (1) detection and quantification of the performance of the DLS and (2) determination of the concordance between actual treatment decisions and those suggested by the algorithm based on DLS quantification of BMs. The first purpose was to evaluate detection and diagnostic accuracy, and we included 100 patients who were negative for BMs. The second purpose was to test whether the treatment suggestions made in patients who were positive for BMs were in accordance with the guidelines. Thus, for the first purpose, we tested a real-world situation of detection of BMs in patients with a clinical suspicion of BMs, with this patient group including patients positive or negative for BM. Then, for the second purpose, we assessed the quality of the treatment suggestions in a group of patients who were all positive for BM.

Most previous similar studies used only GRE CE-T1WI [1, 3–8] and showed false-positive rates higher than the 0.72 per patient found in our study. Because of the high numbers of false positives on GRE CE-T1WI, the next step of introducing DL systems into clinical applications has been limited. Prior research demonstrated the clinical effectiveness of DL using black-blood techniques [13, 21], with one study [13] showing that a dual-enhanced 3D GRE and 3D TSE model had superior sensitivity for detecting small nodules than a 3D GRE-only model. Another study [21] developed a DL system using a volume isotropic simultaneous interleaved bright and black-blood estimation (VISIBLE) sequence that allows for simultaneous acquisition of images with and without blood vessel suppression. The VISIBLE sequence model demonstrated higher sensitivity than radiologists but a greater number of false positives per case, but fewer than a GRE model.

In this study, the false positives were reduced by using a blood vessel suppression technique in turbo-spin echo imaging. A DLS with high quantification performance can help to stratify patients according to risk groups, thus enabling clinical applications in various fields, such as optical coherence tomography of patients with retinal geographic atrophy [22]. Using our system’s good detection and quantification performance, we made a framework that makes decisions that are closely aligned with the clinical decision-making process. This would not only allow clinicians to receive information on the BMs themselves but also provide interpretable numbers and volumes that can easily be visualized and inspected.

Currently, only a limited number of studies have conducted image segmentation for BM. Object detection has been performed by predicting bounding boxes using a “You Only Look Once” (YOLO) model or recurrent CNN, while image segmentation with the prediction of masks has been performed using U-Net and other segmentation networks. When performing object detection, it is important to label the threshold of the bounding boxes as positive or negative according to the ratio of overlap with the ground-truth metastases. In previous studies using object detection for BM, Zhou et al [8] considered a 20% or more overlap as positive, whereas Zhang et al [1] assigned it a 70%. The large difference in threshold values between these two studies shows that this value is highly subjective and ambiguous. Furthermore, because object detection does not involve segmentation, accurate volumetry is impossible, and the technique would be of limited use in a follow-up setting. We overcame the ambiguity of proximity and enabled volumetry by pixel-wise segmentation using U-Net.

There are several limitations to our study. First, this is a retrospective study with a relatively small patient cohort from a single center. External validation is necessary to investigate the model’s robustness. Second, our DLS can underestimate the volume of cystic/necrotic lesions because only the contrast-enhancing portion of rim-enhancing lesions is included in the segmentation. Thus, when a cumulative volume > 15 mL is estimated for a certain patient, the actual lesion volume may be larger if cystic/necrotic masses are present. The RECIST and RANO guidelines state measurement of the maximum diameter, even if the tumor contains cystic/necrotic areas, and we acknowledge that the volume of cystic/necrotic lesions can be underestimated by our DLS. The clinical meaning of this discrepancy needs to be investigated in a future study. Third, the clinical benefit of our DLS needs to be assessed by measuring its impact on clinicians and changes in the long-term outcomes of patients. Treatment decisions for BMs are complex beyond the guidelines, and the real impact on clinical practice should be assessed in a future prospective study.

In conclusion, a deep learning system-based detection and quantification enabled providing the number and volume of brain metastasis at the time of diagnosis. This was translated into a high degree of correlation between a DL-based algorithm for treatment decision and the actual prescribed treatment. A future prospective multicenter study is needed to confirm the clinical adequacy of the system.

### Supplementary Information

Below is the link to the electronic supplementary material.

Supplementary file1 (PDF 743 kb)

## Abbreviations

ALK: Anaplastic lymphoma kinase
ASCO: American Society of Clinical Oncology
ASTRO: American Society for Radiation Oncology
BM: Brain metastasis
CE-T1WI: Contrast-enhanced T1-weighted imaging
DLS: Deep learning system
EGFR: Epidermal growth factor receptor
GRE: Gradient echo
NSCLC: Non-small cell lung cancer
RANO: Response assessment in neuro-oncology
SNO: Society for Neuro-Oncology
SRS: Stereotactic radiosurgery
WBRT: Whole-brain radiotherapy

## Declarations

### Guarantor

The scientific guarantor of this publication is Ho Sung Kim.

### Statistics and biometry

No complex statistical methods were necessary for this paper.

### Study subjects or cohorts overlap

Study subjects or cohorts have not been previously reported.

### Methodology

• Retrospective

• Diagnostic or prognostic study

• Performed at one institution
