# Supplementary material for: Deep learning-based detection and quantification of brain metastases on black-blood imaging can provide treatment suggestions: a clinical cohort study
Source: Eur Radiol. 2023 Sep 2;34(3):2062–71. doi: 10.1007/s00330-023-10120-5 (PMC10873231; doi:10.1007/s00330-023-10120-5)
Supplement: Supplementary file 1 — Supplementary file1 (PDF 743 kb) [file 330_2023_10120_MOESM1_ESM.pdf]

# Deep learning-based detection and quantification of brain metastases on black-blood imaging can provide treatment suggestions: A clinical cohort study

## Electronic Supplementary Material (ESM)

**I. Supplementary Materials:** *MRI acquisition protocols, DLS for detection and quantification of BM*

**II. Supplementary Materials:** *Discrepancies between actual clinical decisions and DLS-based suggestions*

**III. Supplementary Table 1.** Distribution of brain metastases in the training set

**IV. Supplementary Table 2.** Performance of deep learning system in the training set

**V. Supplementary Table 3**. Frequent patterns of false-positive and false-negative findings

**VI. Supplementary Table 4.** Cases showing discrepancies between the actual clinical decision and DLS based treatment suggestion

**VII. Supplementary Figure 1.**

**VIII. Supplementary Figure 2.**

**IX. Supplementary Figure 3.**

**X. Supplementary Figure 4.**

*Eur Radiol (2023) Jeong H, Park JE, Kim N, Yoon SK, Kim HS*

# I. Supplementary Materials

*MRI acquisition protocols*

A black-blood imaging sequence was added to our routine BM protocol in accord with the consensus recommendations for MRI of brain metastasis[1]. Five minutes after administration of gadolinium-based contrast (0.05 ml/kg gadoterate meglumine; Dotarem; Guerbet), gradient-echo(GRE) CE-T1WI and black-blood CE-T1WI were acquired. The imaging parameters for the GRE CE-T1WI were repetition time (TR)/echo time (TE), 9.8/4.6 ms; flip angle, 8°; field of view, 24 cm; section thickness, 1 mm; acquisition matrix, 256 × 256; reconstructed matrix, 512 × 512; and acquisition time, 3 minutes. The parameters for the black-blood CE-T1WI were TR/TE, 600/28.4 ms; flip angle, 90°; field of view, 24 cm; section thickness, 1 mm; acquisition matrix, 256 × 256; reconstructed matrix, 512 × 512; and acquisition time, 4 minutes 43 seconds. The improved motion-sensitized driven-equilibrium prepulse consisted of one 90° excitation pulse, two 180° refocusing pulses, and one 90° excitation pulse with motion-sensitized gradients between radiofrequency pulses. The duration between the two 90° pulses was 28.3 ms, and the flow velocity encoding for gradient pulses was 3 cm/s.

*DLS for detection and quantification of BM*

The GRE CE-T1WI and black-blood CE-T1WI were coregistered using rigid transformations with six degrees of freedom in SPM (version 12, www.fil.ion.ucl.ac.uk/spm/). Skull stripping was performed using an algorithm optimized for heterogeneous MRI data with diverse pathology or post-treatment changes (https://github.com/MIC-DKFZ/HD-BET). The lesion segmentation model was implemented using nnUnet, a 3D U-Net-based method (https://github.com/MIC-DKFZ/nnUNet) [2] in the PyTorch package version 1.1 in Python 3.7 (www.python.org). In our model training, a GRE CE-T1WI and black-blood CE-T1WI image

pair were fed into the model as input. A full-resolution 3D model was developed because it was expected that such a model would show optimum performance with the small simple-shaped lesions characteristic of BM and the possibility of multiple lesions within a single image. The training data were augmented with rotation, gamma, scaling, elastic deformation, and mirror transformations. The model training details included dice and cross-entropy loss function, Adam optimizer with learning rate weight decay, learning rate (initial) 0.0003, batch size 2, and patch size 128 × 128 × 112. Model training was conducted with an NVIDIA TITAN RTX 24 GB GPU with CUDA version 10.0, and the model performance converged after 507 epochs over 2.5 days.

## II. Supplementary Materials

*Discrepancies between actual clinical decisions and DLS-based suggestions*

Group A was indicated for follow-up, group B was limited to patients with BMs indicated for surgery or SRS, and group C was patients with extensive BMs suggested for chemotherapy or WBRT by the DLS. The case showing the greatest discrepancy was a patient who was suggested as belonging to group A by the DLS, whereas the patient actually received WBRT. The patient had pancreatic cancer and two BMs in the cerebellum with a total volume of 27 $mm^3$. He also had disseminated bone metastasis in the whole spine. The clinician initially planned SRS for BMs; however, the delay for the scheduled SRS was considered too long, and radiation therapy was performed simultaneously on the spine and cerebellum.

Four patients were suggested as group B by the DLS but did not receive any treatment. Of these, one patient had primary lung cancer and five BMs with a total volume of 155 $mm^3$. The burdens from primary lung cancer and liver metastasis were high at the same time. Although she needed treatment for BM, the treatments for lung and liver lesions were given clinical priority, and the patient was initially followed-up, with SRS for BM then being performed 7 months later. The other three patients had NSCLC with PDL1 expression and underwent pembrolizumab treatment as part of a clinical trial.

The last two cases (patients 20 and 21) in the table were considered likely to benefit from DLS-based treatment. Although they had multiple lesions, only selective stereotactic radiosurgery was performed on a few lesions, and follow-up MRI showed an increase in the size and number of the other lesions. We believe that if whole brain RT had been performed, as suggested by the DLS, different results may have been obtained.

1. Kaufmann TJ, Smits M, Boxerman J et al (2020) Consensus recommendations for a standardized brain tumor imaging protocol for clinical trials in brain metastases. Neuro Oncol 22:757-772
2. Isensee F, Schell M, Pflueger I et al (2019) Automated brain extraction of multisequence MRI using artificial neural networks. Hum Brain Mapp 40:4952-4964

**Supplementary Table 1.** Clinical characteristics and distributions of brain metastases in the training set

| Parameter | |
|---|---|
| Number of patients (with vs without BM) | 193 (93 vs 100) |
| BM Number | 864 |
| Average number of BM for each patient | 9.46 ± 14.4 |
| Patients with >10 numbers of BM | 20 (21.5%) |
| Size (mm) of BM | |
| Mean ± SD | 20.4 ± 81.2 |
| BM numbers <10 mm | 227 (26.2%) |
| Characteristics of BM patients | |
| Age (years)* | 64.3 ± 9.5 |
| Gender (male:female) | 113:80 |
| Cancer type (%) among positive BM | |
| Lung | 86 (44.6) |
| Breast | 3 (1.6) |
| Colon | 1 (0.1) |
| Renal | 2 (0.1) |
| Other | 1 (0.1) |
| Systemic chemotherapy (%) | 49 (25.4) |
| Immunotherapy (%) | 12 (6.2) |

**Supplementary Table 2.** Performance of deep learning system in the training set

| Performance | |
|---|---|
| Sensitivity | 87.7% [758/864] |
| Positive predictive value | 80.6% [758/940] |

**Supplementary Table 3.** Frequent patterns of false positive and false negative findings

| | |
|---|---|
| False positive findings | |
| Pattern 1 | Vascular enhancement |
| Pattern 2 | Incidentally found extra-axial mass |
| Pattern 3 | Rim enhancing lesion counted as multiple lesions |
| Pattern 4 | Others (large solid enhancing mass counted as multiple enhancing lesions, dural enhancement) |
| False negative findings | |
| Pattern 1 | Tiny faintly enhancing lesions not segmented |
| Pattern 2 | Poorly enhancing lesion with iso signal intensity compared with white matter |
| Pattern 3 | Tiny enhancing lesion in the brainstem not segmented |

**Supplementary Table 4.** Cases showing discrepancies between the actual clinical decision and DLS-based treatment suggestion

| Patient | Ground truth lesion count | DLS-based lesion count_ | Ground truth volume ($mm^3$) | DLS-based volume ($mm^3$) | Actual clinical decision | DLS-based treatment suggestion | Comment |
|---|---|---|---|---|---|---|---|
| 1 | 1 | 1 | 25 | 26 | B | A | Clinicians decided to perform stereotactic radiosurgery when other aspects were considered, despite the lesions being small. |
| 2 | 1 | 1 | 6 | 5 | B | A | |
| 3 | 2 | 1 | 11 | 8 | B | A | |
| 4 | 5 | 2 | 50 | 24 | B | A | |
| 5 | 2 | 1 | 27 | 18 | C | A | The patient also had disseminated bone metastasis in the entire spine. The patient had symptoms so the clinician initially planned SRS for BMs. However, SRS was expected to be too delayed, and radiation therapy was performed simultaneously on the spine and cerebellum. |
| 6 | 1 | 1 | 63 | 66 | A | B | Disappeared or no change on 2-month to 1-year follow up MRI |
| 7 | 3 | 3 | 53 | 46 | A | B | |
| 8 | 1 | 1 | 72 | 71 | A | B | |

| | | | | | | | |
|---|---|---|---|---|---|---|---|
| 9 | 5 | 4 | 155 | 126 | A | B | The burdens of primary lung cancer and liver metastasis were increasing at the same time. The treatment for lung and liver lesions was given clinical priority, and SRS for BM was performed 7 months later. |
| 10 | 9 | 5 | 383 | 241 | C | B | NSCLC patients with PD-L1 expression, who were treated with pembrolizumab |
| 11 | 4 | 4 | 173 | 178 | C | B | |
| 12 | 3 | 7 | 51 | 72 | C | B | |
| 13 | 7 | 9 | 73 | 58 | C | B | The patients had not only brain metastasis but also leptomeningeal seeding or bone metastasis, and were therefore treated by WBRT or chemotherapy |
| 14 | 6 | 5 | 54 | 55 | C | B | |
| 15 | 9 | 8 | 51 | 67 | C | B | |
| 16 | 23 | 24 | 739 | 628 | B | C | Selective stereotactic radiosurgery for a few lesions. Other untreated lesions showed no change or decreased size on follow up MRI |
| 17 | 24 | 24 | 421 | 411 | B | C | |
| 18 | 10 | 12 | 2605 | 2621 | B | C | |
| 19 | 1 | 7 | 39794 | 15803 | B | C | |
| 20 | 39 | 41 | 8899 | 9496 | B | C | Selective stereotactic radiosurgery for a few lesions. Increased size and numbers of other lesions found on follow up MRI |
| 21 | 19 | 17 | 741 | 637 | B | C | |

## Supplementary Figures

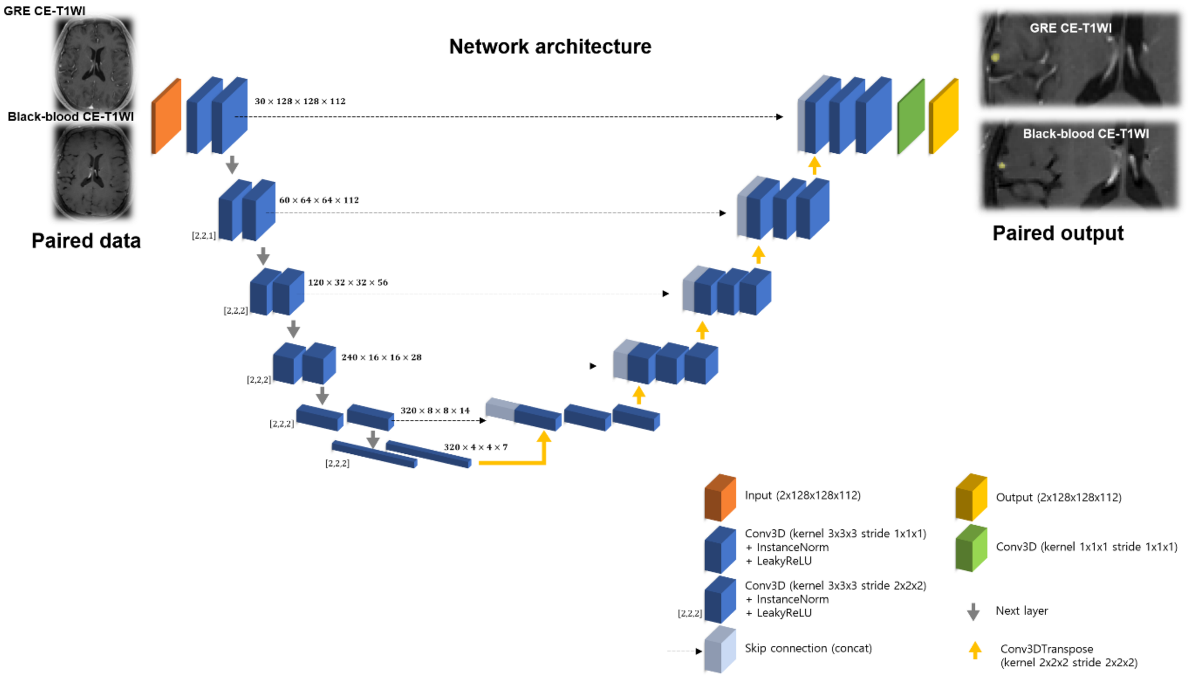


**Supplementary Figure 1.** The network design used in this study. The trained U-Net architecture and more detailed information such as feature map size, kernel, and strides. Since the input image size varies for each case, several patches are generated using a sliding window approach. Model prediction results for each patch overlap by half of the size of a patch and are aggregated to generate the final lesion mask.

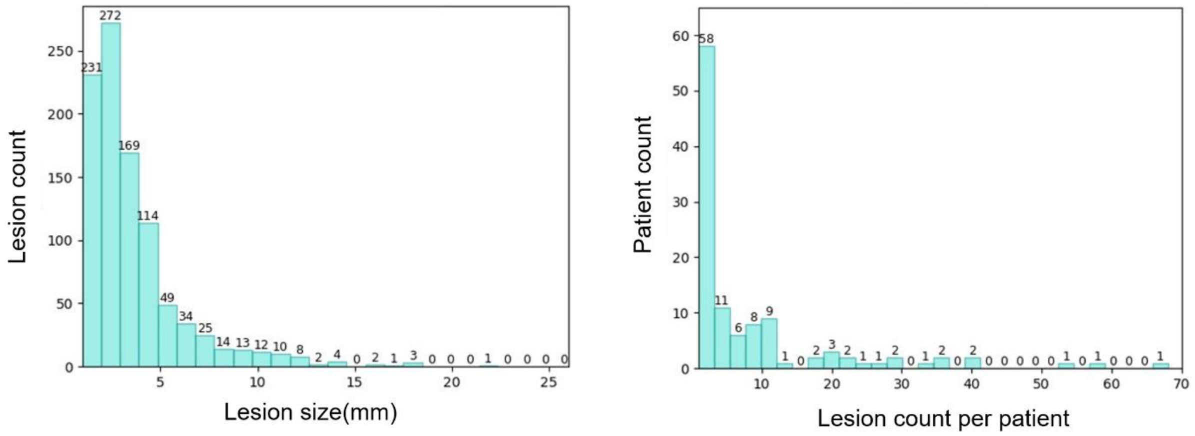


**Supplementary Figure 2**. Bar charts show distribution of brain metastasis size and count in clinical cohort.

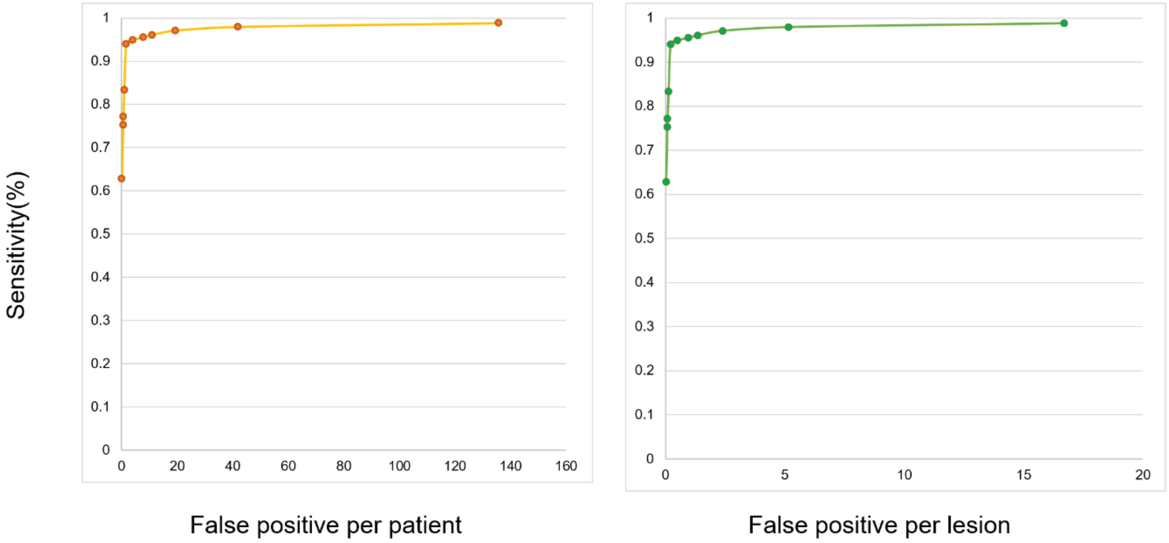


**Supplementary Figure 3**. Free response receiver operating characteristic curve for all patients and all lesions are shown.

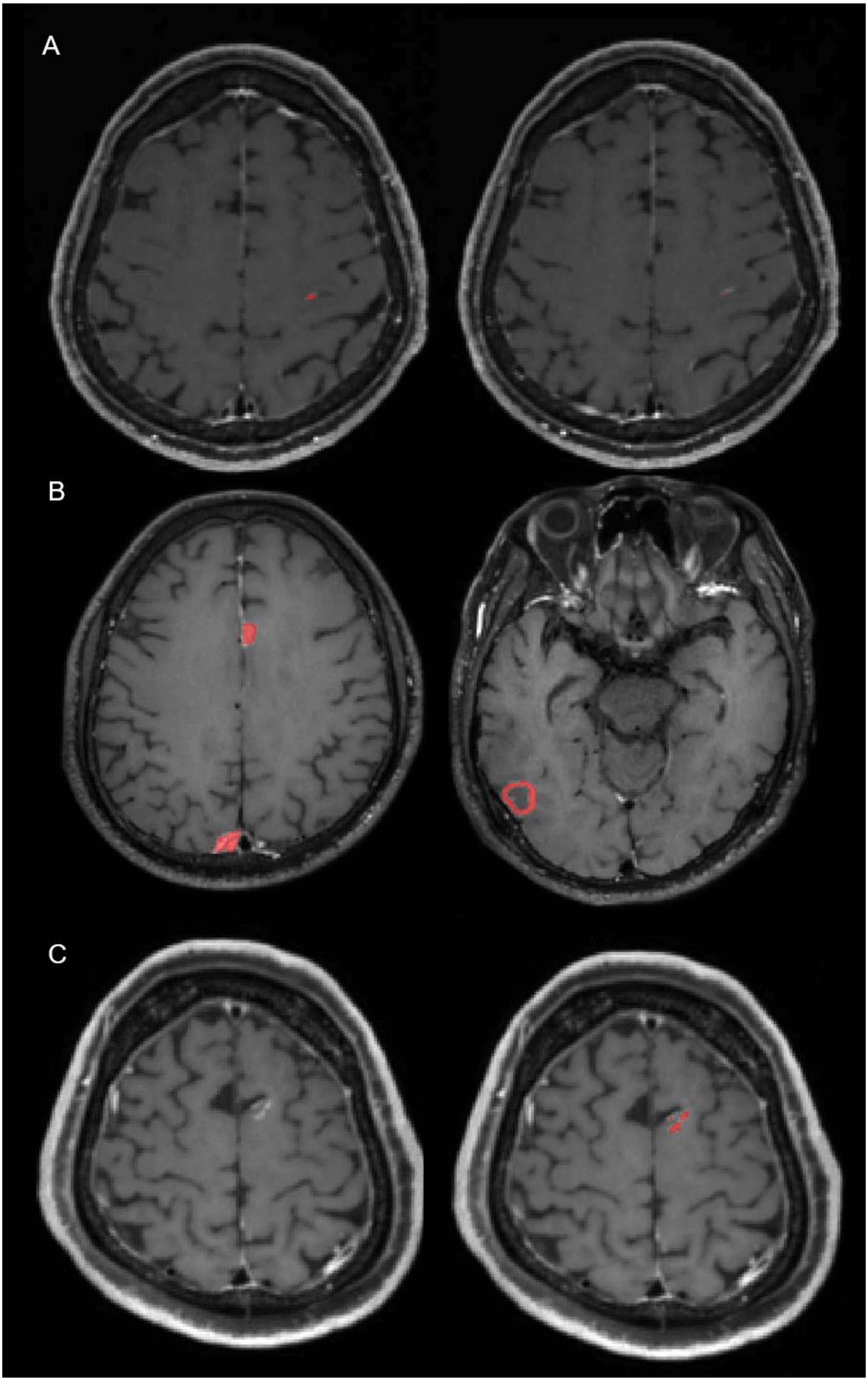
A
B
C

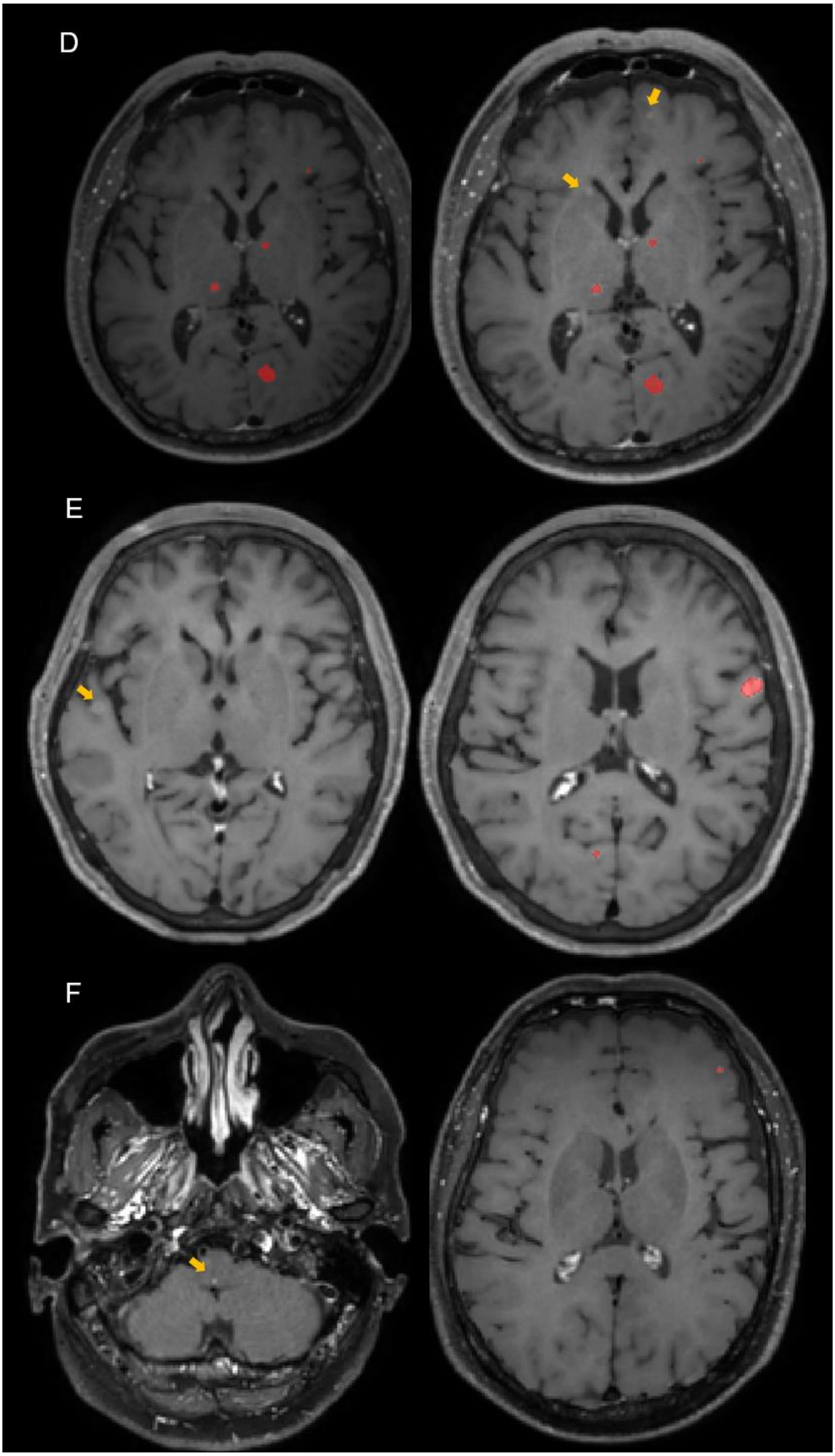

D
E
F

**Supplementary Figure 4**. Frequent patterns of false positive (A-C) and false negative (D-F) findings: false positive findings occurred in (A) vascular enhancement, (B) incidentally found extra-axial mass, (C) rim enhancing lesion counted as multiple lesions. False negative findings (orange arrows) occurred in (D) tiny faintly enhancing lesions, (E) poorly enhancing lesions (isosignal intensity), and (F) an enhancing lesion in the brainstem.
